# Exploring *Cuscuta epithymum’s* Effect on Neuroinflammation, Tyrosine Kinase Activity and Macrophage Counts in Spleen and Liver: Revealing Their Roles in Stress Responses

**DOI:** 10.34172/apb.025.45554

**Published:** 2025-08-11

**Authors:** Leila Ghassemifard, Narjes Khavasi, Ehsan Saboory, Fatemeh Madani, Saeed Sardari

**Affiliations:** ^1^Department of Persian Medicine, School of Medicine, Zanjan University of Medical Sciences, Zanjan, Iran; ^2^Department of Addiction Studies, School of Medicine, Zanjan University of Medical Sciences, Zanjan, Iran; ^3^Zanjan Metabolic Diseases Research Center, Health and Metabolic Diseases Research Institute, Zanjan University of Medical Sciences, Zanjan, Iran; ^4^Department of Nanotechnology, School of Advanced Technologies in Medicine, Tehran University of Medical Sciences, Tehran, Iran

**Keywords:** *Cuscuta epithymum*, Stress, Tyrosine kinase, Macrophage, Megakaryocyte, TNF-α, IL-1β, Persian medicine

## Abstract

**Purpose:**

Chronic stress usually causes immunosuppression, activates tyrosine kinase (TK), and increases inflammatory responses. Based on Persian medicine, the spleen is crucial for the immune system and stress response. Cuscuta epithymum (CE) contains antioxidant properties and is beneficial to the immune system.

**Methods:**

In this experimental study, 28 male and 56 female rats were randomly divided into four groups and exposed to stress from restraint. Simultaneously, Cuscuta’s extract was given to the other two groups while normal saline was given to the control and stressed rats. Four different coupling combinations were created by mating control and experimental rats: McFc, MsFs, McFc+EX, and MsFs+EX (M: male, F: female, C: control, S: stress, and EX: extract). The TK level, megakaryocyte, and macrophage cell number in the liver and spleen were then assessed after certain parents and male pups were dissected on postnatal day (PND) 25. Western blot analysis was used to measure the brain’s quantitative levels of TNF-α and IL-1β protein expression.

**Results:**

Rats under stress had much higher levels of TK and macrophage cells in their liver and spleen tissues than the other rats, while the stress+CE group had significantly lower levels. While megakaryocyte cells increased in CE-treated animals, they dramatically declined in the stress group. The brain homogenate’s TNF-α and IL-1β levels were considerably lowered by *Cuscuta* extract.

**Conclusion:**

Our study showed the significant role of the *Cuscuta* in decreasing the adverse effects of stress on the liver and spleen immune system, as well as a remarkable anti-neuroinflammatory effect.

## Introduction

 In contemporary society, stress is an inevitable component of existence, and it results in changes to the immune and sympathetic nervous systems, which exacerbate liver and splenic diseases.^1^ This can result in the disruption of a host’s defenses. The activation of immune and inflammatory responses can be induced by a variety of stressors, including immobility stress, which causes oxidative stress and inflammation.^2,3^ This results in the stimulation of spleen and liver macrophage cells, as well as the activation of pro-inflammatory cytokines like TNF-α and IL-1β.^4,5^ Prenatal stress (PS) or pre-pregnancy stress in rodents resulted in molecular and structural alterations that are transmitted to the subsequent generation, as well as an impairment in the feedback inhibition of hypothalamic-pituitary-adrenal (HPA) axis activity.^6,7^ Therefore, it is possible to note that the activation of tyrosine kinase (TK), followed by the activation of macrophages, thus, TK inhibitor drugs, such as Imatinib and Sunitinib that prevent the production of inflammatory and pro-inflammatory cytokines should be used. But, the chemical compounds and drugs that currently exist to inhibit TK have caused various side effects, including serious toxic effects on the heart, lungs, liver, kidneys, thyroid, etc.^8,9^ Some herbal medicines have a great effect in treatment with fewer side effects, and it showed a high antioxidant effect. *Cuscuta epithymum* (CE) is one of the plants that inhibits TK^10^ and regulates the activity of immune system.^11^ CE is one of the most important plants of *Cuscutaceae* family that parasitically live on other plants, and contains a high content of flavonoids, quercetin, kaempferol, coumarins and lignin glycosides,^12,13^ also it can be used as a plant with anti-depressant and anti-convulsant effects.^14^ In Persian medicine, it is used for treatment of headache and obsessive-compulsive disorder, insanity,^15^ and liver diseases^16^; Consequently, we determined to examine the effects of CE on TK levels, macrophage and megakaryocyte numbers in the liver and spleen, and TNF-α and IL-1β levels in the brain of two generations, in light of the immune system effects,^17^ inhibition of TK, and the anti-inflammatory effects.

## Material and Methods

###  Subject

 This experimental study used 84 rats (56 females and 28 males), each aged 8 weeks. To reduce the rat population, each set of male rats was mated once with both the stressed and control female rats. Upon arrival at the animal home of Zanjan University of Medical Sciences, the rats were maintained for a period of two weeks to facilitate acclimation. The sexual cycle of female rats was synchronized by proximity to sexually restricted males for three days. Then, males and females were allocated into four groups (each n = 7 in males and n = 14 in females): 1. Control (neither stress nor CE extract), 2. CE Extract (just received extract), 3. Stress (just received restraint stress), 4. Stress + CE extract (received both stress and extract). The rats in stress groups (Stress and Stress + CE) were subjected to immobility stress: Male rats were subjected to 2 hours of daily restraint stress for 50 days, while female rats underwent the same protocol for 15 days. CE-treated rats were orally administered 100 mg/kg of CE extract in each session at 8 to 10 AM. At the end of stress and treatment phase, the rats were mated by caging a male and a female per cage. The mating cage was placed in a dark and quiet place at 8:00 AM until next morning for 24 h. Control and experimental rats were mated so that to make four kinds of coupling pairs as follows McFc, MsFs, McFc + EX, MsFs + EX (M: male, F: female, C: control, S: stress, EX: extract). Immediately after mating, male rats were sacrificed under isoflurane anesthesia and their brain, spleen, and liver were dissected to determine some variables that will be explained later.

 The females were segregated from the males by a vaginal plaque and housed in groups of four under a consistent 12-hour light and dark cycle until parturition. Following parturition, a mother and her puppies were housed in a separate cage until experimental day on postnatal day (PND) 25. Subsequently, male progeny was subjected to dissection under general anesthesia with isoflurane on postnatal day 25, and their spleen and liver were removed for TK analysis with fluorescence techniques. The quantity of megakaryocytes and macrophages in the spleen and liver was assessed using hematoxylin and eosin (H&E) staining. Simultaneously, the brain was dissected to quantify TNF-α and IL-1β protein levels using western blotting. The trials were conducted on male progenitors as well.

###  Preparing extract and administration

 Percolation method was used to extract CE (Herbarium code 5501) plant extract. For this purpose, CE plant was ground and sieved with two standard sieves (160 and 250 meters). Then, 2000 g of ground plant was poured into the percolator and then 70% ethanol was added to this powder. The solvent of obtained extract was separated by a rotary evaporator at a temperature of 45-50 °C (RV; 05B, IKA, Germany). The drying process continued until the weight was constant. The method was repeated in three 24-hour periods. The extract was then filtered, frozen, and lyophilized in a water bath under vacuum pressure (model: FD-5N; Eyela, Tokyo).^18^ The acquired hydroalcoholic extracts were stored in a refrigerator at 4 °C until used.^5,19^ On the day of administration, the necessary quantity of extract was combined with normal saline to achieve the required concentration.

###  Restraint stress procedure 

 Immobility stress was applied to the rats based on Nakhjiri et al.^20^ In brief, the protocol involved the transport of rats to the experimental room, in which they were kept in a restrainer. The rats remained inside the confinement chamber (cylindrical plastic enclosure 16 cm long and 6 cm in diameter) once a day for 2 hours.

###  Hematoxylin and eosin staining

 H&E staining was used to identify the histopathological lesion. Brain Tissue was fixed with 4% paraformaldehyde, embedded in paraffin and cut into slices. The histological changes of brain tissue were observed by Tissue FAXS (Tissue Gnostics, Vienna, Austria) at 400 × magnification.^21^

###  Western blotting

 Protein expression of brain TNF-α and IL-1β was determined via normal Western blot analysis. In summary, the brain tissue from four groups of rats, at a concentration of 500 mg of tissue per 10^6^ cells, was homogenized in 500 µL of lysis solution. The samples were then centrifuged at 12000 rpm for 10 minutes at 4 °C using an Eppendorf 5415 R centrifuge. The protein-containing clear liquid (supernatant) was removed and kept at -20 °C. The protein concentration was then determined using the Brad-Ford technique. The supernatant samples were denatured by heating at 95 °C in an SDS sample buffer before being placed onto 4-20% Mini-Protean TGX Precast gels and then electronically transferred to a PVDF membrane. The membranes were treated with rat primary antibodies (1:300, β-actin for equal loading: Sc47778, TNF-α: Sc130349, IL-1β: SC2357). Following thorough washing, the membrane was treated with the horseradish peroxidase-linked anti-rabbit secondary antibody (1:1000; Sc2357) and proved positive. The ECL advanced reagents kit, which included non-fat milk and Reagents A and B in a 1:1 ratio, was used to identify the appropriate protein band. The film was then scanned, and the optical densities of protein bands were calculated using Image J software.^20^ Finally, we used the following approach to normalize the raw data: The target density for each sample was divided by the target density for the control loading (beta actin). We split the collected data depending on the control’s TARGET DENSITY/LOADING number.^22^

###  Immunofluorescence staining

 During the dissection of animals, liver and spleen tissues were harvested and immersed in 10% formaldehyde for fixation. The tissues underwent a two-step fixation process as follows: 1. De-paraffinize sections: xylene and ethanol. 2. Hydrate step and blocked it using paraffin. After that, the slides were recovered in citrate buffer. The samples were blocked for two hours at room temperature using 1% BSA, and then they were treated for two hours at 28°C with primary antibodies (1:100 in Dako antibody diluent). As the secondary antibody, FITC-conjugated goat anti-mouse IgG H&L (ab6785) (1/800) was used. After that, DAPI was used to mark the cell nuclei, giving them a blue hue (Sigma-Aldrich). Thermo Fisher Scientific, Waltham, MA, USA, provided the ProLong Gold Anti-fade reagent for mounting the cells. The slides were cleaned in TBS plus 0.03% Triton X-100 in between each step. A fluorescent microscope (Microscope Camera: Olympus DP72, Mshot, China; microscope: BM-600 LED EPI, fluorescent and light microscope, Germany-AXIOM) and 2012 software (Carl Zeiss) were used to capture the images.^23^

###  Statistical analyses

 Data analysis was conducted using SPSS version 22.0 and GraphPad Prism version 9.00. The Kolmogorov-Smirnov test demonstrated that the data distribution was normal for each parameter. A one-way ANOVA and Tukey’s pairwise comparison tests were then conducted. The graphs were made using GraphPad Prism and GraphPad Prism 9. The results were presented as mean ± SEM when shown as a bar graph or as single data points with the mean in a scatter dot plot. The *P *values were presented as follows: * *P* < 0.05, ** *P* < 0.01, and *** *P* < 0.001.

## Results

###  Percentage of TK expression in spleen

**Figure 1:**
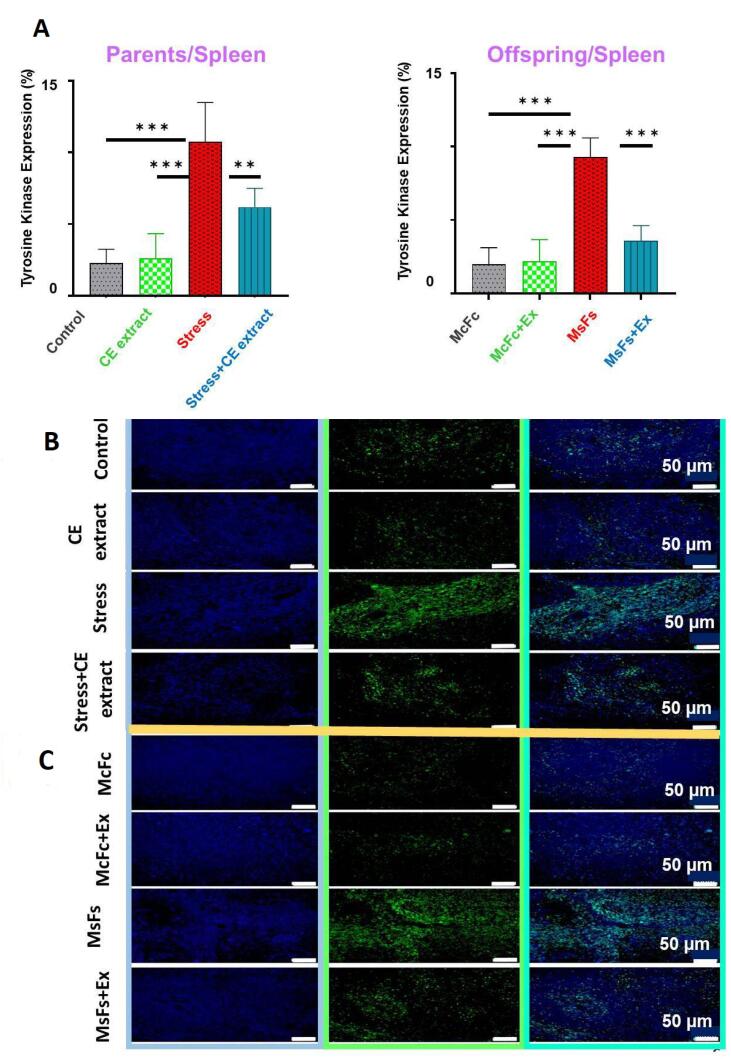
Effect of *Cuscuta epithymum* (CE) extract on spleen TK expression (%) in male parents subjected to immobility stress and their male offspring. Data were analyzed using one-way ANOVA followed by Tukey’s post hoc test (n = 8 for both parents and offspring); *P* < 0.01**, *P* < 0.001*** (Panel A). Protein tyrosine kinase levels were assessed by immunofluorescence staining in parents ( × 200, Panel B) and offspring ( × 200, Panel C)

 The results of immunofluorescence analysis indicate that the percentage of TK expression in the spleen of male parents and their male offspring in the stress group was significantly higher than those of the extract and control groups (*P < 0.001*). Thus, in the stress + CE extract group, the percentage of TK expression decreased in male parents (*P* < 0.01), and in offspring (*P* < 0.001) compared to the stress group (Figure 1A, 1B, and 1C).

###  Percentage of TK expression in liver tissue

**Figure 2:**
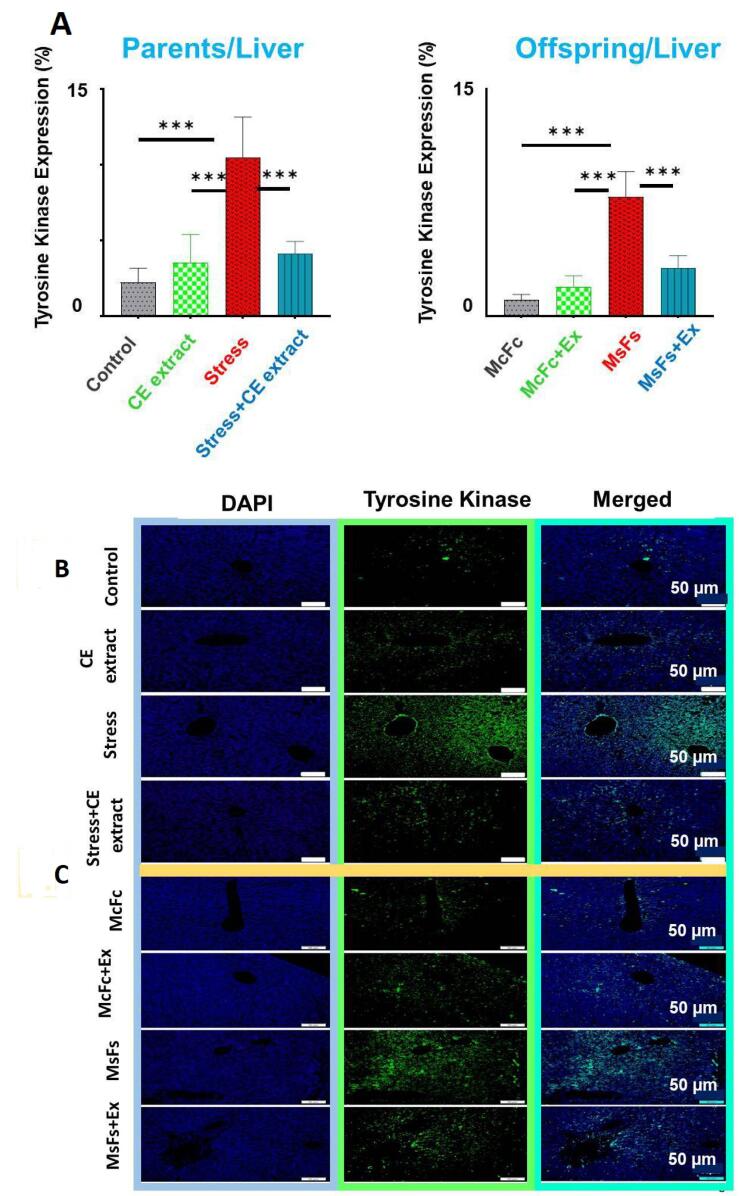
Effect of *Cuscuta epithymum* (CE) extract on liver TK expression (%) in male parents subjected to immobility stress and their male offspring (n = 8 for both parents and offspring); *P* < 0.001*** (Panel A). Protein tyrosine kinase levels were assessed by immunofluorescence staining in parents ( × 200, Panel B) and offspring ( × 200, Panel C)

 The percentage of TK expression in the parental and offspring stress groups was significantly higher than that in the extract, control, and CE + stress groups, according to the quantitative analysis of liver tissue (*P* < 0.001). The TK data were analyzed using Tukey post hoc testing and one-way ANOVA (Figure 2A, 2B, and 2C).

###  The quantity of megakaryocytes and macrophages in spleen tissue

**Figure 3:**
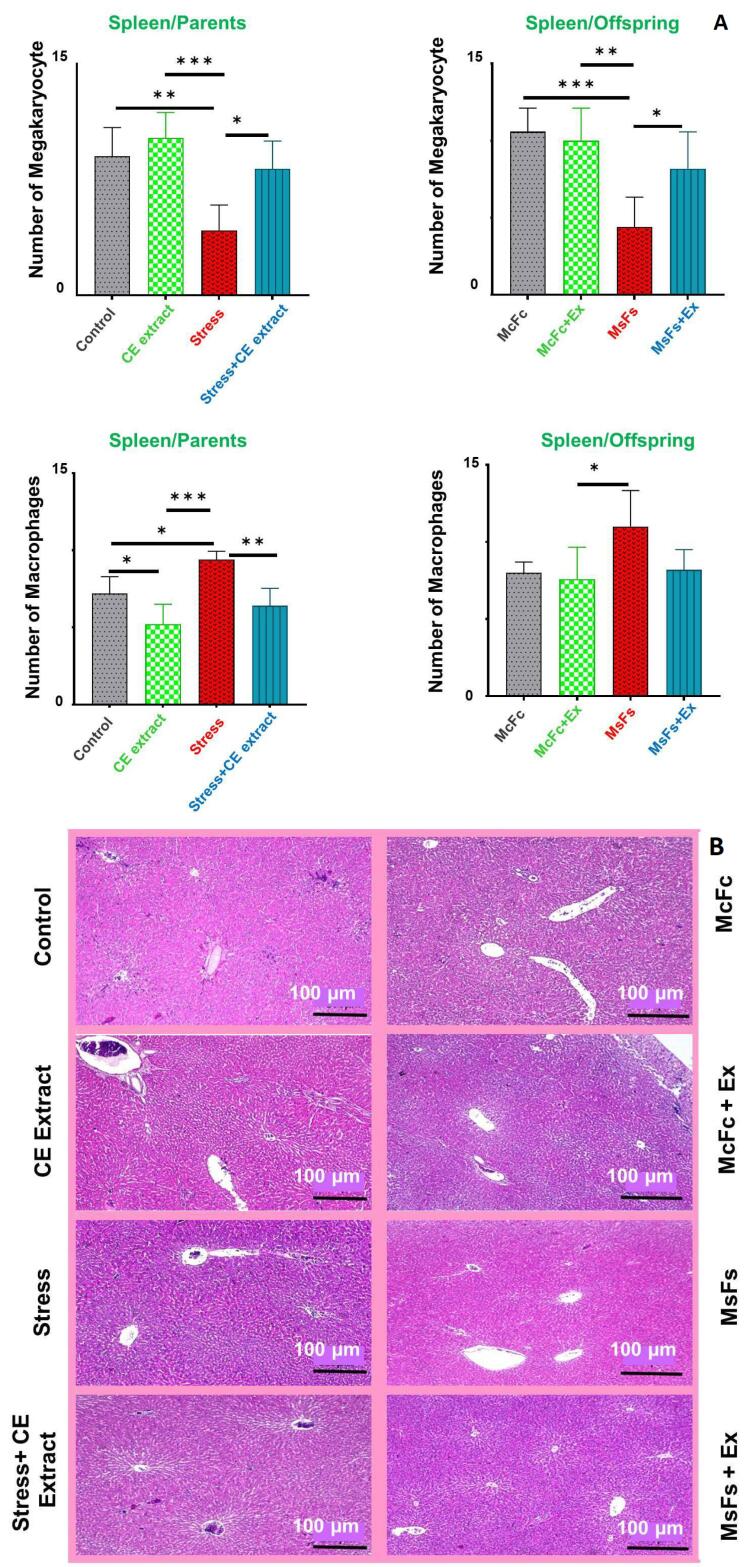
Effect of *Cuscuta epithymum* (CE) extract on the number of megakaryocyte and macrophage cells in spleen of male parents subjected to immobility stress and their male offspring. Data were analyzed using one-way ANOVA followed by Tukey’s post hoc test (n = 8 for both parents and offspring); *P* < 0.05*, *P* < 0.01**, *P* < 0.001*** (Panel A). Optical microscope image of the spleen showing megakaryocyte and macrophage cells in parents (left) and offspring (right); H&E staining, × 40 (Panel B)

 The splenic megakaryocyte count was considerably lower in the stress group (parents and children) than in the control and extract-receiving groups (*P* < 0.01; *P* < 0.001). Megakaryocyte counts were significantly higher in the CE extract-treated stress group than in the non-CE extract group (*P* < 0.05). When compared to their respective controls, the stress groups of both parents (*P* < 0.01) and offspring (*P* < 0.05) showed a significant increase in macrophage count. Additionally, there were no notable differences between the parents and children in the extract-treated stress group and either the extract group or the control group (Figure 3).

###  The quantity of megakaryocytes and macrophages in liver

**Figure 4:**
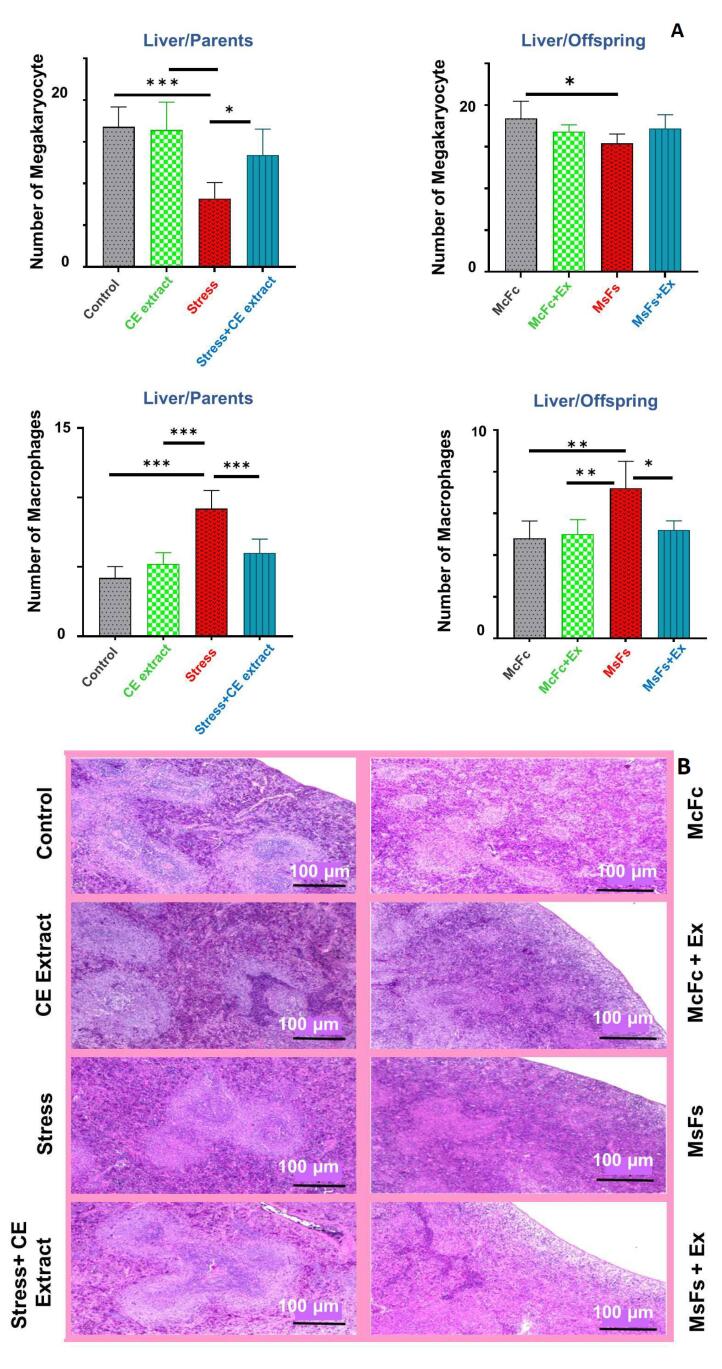
Effect of *Cuscuta epithymum* (CE) extract on the number of megakaryocytes and macrophage cells in liver of male parents subjected to immobility stress and their male offspring. Data were analyzed using one-way ANOVA followed by Tukey’s post hoc test (n = 8 for both parents and offspring); *P* < 0.05*, *P* < 0.01**, *P* < 0.001*** (Panel A). Optical microscope image of the liver showing megakaryocyte and macrophage cells in parents (left) and offspring (right); H&E staining, × 40 (Panel B)

Figure 4 illustrates that the stress + CE extract group had significantly more megakaryocytes in the male parents’ livers than the stress group (*p* < 0.05). The megakaryocyte numbers of the MsFs + extract offspring, however, did not significantly alter. Compared to the stress group, the stress + CE extract group’s liver tissue had significantly fewer macrophages (*p* < 0.001), and the offspring likewise shown a decrease (*P* < 0.05). Remarkably, the mean results for the stress + CE extract group were comparable to those of the other groups, indicating that neither the extract group nor the control group significantly altered the behavior of the parents or children.

###  Protein levels of brain TNF-α

**Figure 5:**
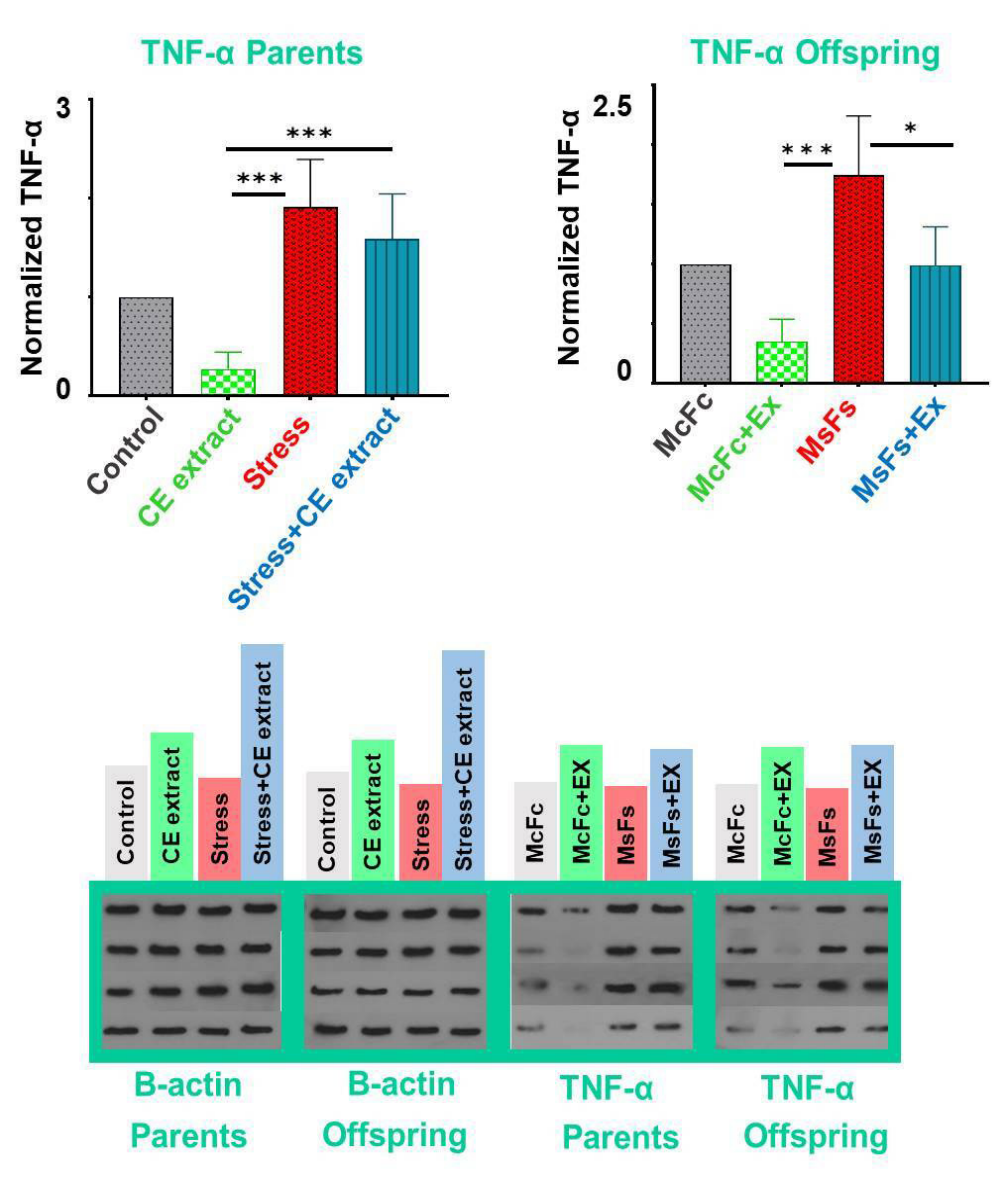
Effect of *Cuscuta epithymum* (CE) extract on TNF-α protein levels in the brains of male parents subjected to immobility stress and their male offspring. Data were analyzed using one-way ANOVA followed by Tukey’s post hoc test (n = 8 for both parents and offspring). A marked increase (*P* < 0.001***) was observed in the stress group compared to the CE-treated group. In the offspring stress group receiving CE extract (MsFs + EX), TNF-α protein levels were significantly reduced compared to the stress-only group (MsFs). * indicates *P* < 0.05; *** indicates *P* < 0.001

 Western blot examination revealed that the stress group’s brains had significantly higher amounts of TNF-α protein than the control group’s (*P* < 0.05) for both parents and male offspring. Additionally, there were no discernible differences between the offspring of the MsFs group and the MsFs + EX group, nor between the stress + CE extract group and the control group (Figure 5).

###  Protein levels of IL-1β in brain of male rats and their male offspring

**Figure 6:**
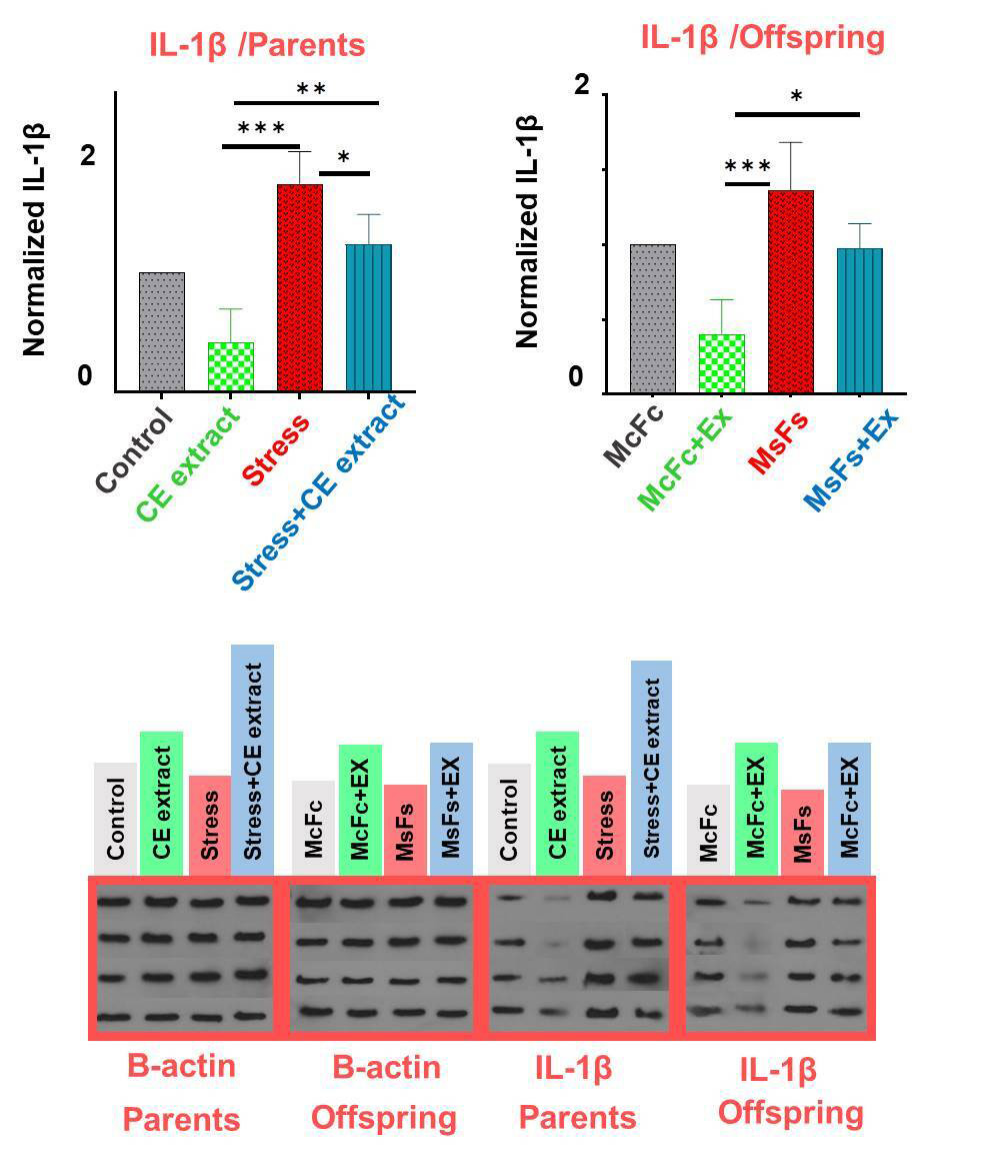
Effect of *Cuscuta epithymum* (CE) extract on IL-1β protein levels in the brains of male parents subjected to immobility stress and their male offspring. Data were analyzed using one-way ANOVA followed by Tukey’s post hoc test (n = 8 for both parents and offspring). A notable increase (*P* < 0.001) was observed in the stress group compared to other groups. In the stress + CE extract group, IL-1β protein levels in parents were significantly reduced (*P* < 0.05). However, neither the stress + CE extract group nor the MsFs + EX group showed a significant difference compared to their respective control groups (*P* < 0.05*, *P* < 0.01**, *P* < 0.001***)

 Western blot analysis showed a significant increase in IL-1β levels in the brains of parents and male offspring in the stress group compared to the control group (*P* < 0.01); CE extract treated rats showed remarkable difference with stressed rats; for more details see Figure 6.

## Discussion

 The liver and spleen are essential components of the immune system, and immobilization stress has a substantial impact on their function.^24-26^ The study’s findings provide strong proof that the immune system is significantly impacted by ongoing immobilization stress. In particular, it increased macrophage numbers and TK activity while decreasing the number of megakaryocytes in the liver and spleen. Both generations of stressed rats had significantly higher levels of pro-inflammatory substances in their brains, such as TNF-α and IL-1β. Additionally, the CE extract demonstrated a noteworthy capacity to mitigate the adverse effects of stress. In the context of CE extract on stress, it was reported that CEextract showed an ability to mitigate the adverse effects of stress; Xia et al demonstrated a significant increase in the expression of TK in the liver and spleen of stressed subjects across two generations.^27^ Hu et al conducted an in-vivo protein kinase assay to examine the expression of protein kinase in various tissues under thermal stress. They reported a substantial activation in the heart, liver, and kidney as a result of the activity of kinases, which is produced by the phosphorylation of proteins in the tissues.^28^ However, CE plant is rich in antioxidants, particularly flavonoids,^29,30^ which caused the dephosphorylation and inhibition of the TK, and then regulated the activity of the immune system.^31^

 The liver and spleen of both parents and children that were subjected to immobility stress showed a substantial decrease in megakaryocytes and an increase in macrophages in the present study. Conversely, rodents that were treated with CE extract exhibited the opposite results. This is consistent with the results of Rajaee et al, who reported a substantial decrease in megakaryocyte counts in the stressed group, despite the absence of any significant changes in macrophage cell counts. In all the parental and offspring groups, CE extract reduced the alterations in immunological and neuroinflammatory markers brought on by stress.^32^ Consequently, it was shown that CE strengthened the immune system, promoted anti-fatigue qualities, increased spleen weight in immature mice, and enhanced tolerance to hypoxic circumstances (stress).^33,34^ Among the main components that give CE its ability to improve antioxidant activity and immunological function are polysaccharides, sterols, and kaempferol.^35^

 Long-term stress can disrupt HPA axis, resulting in systemic pro-inflammatory conditions. This disruption leads to the overproduction of pro-inflammatory cytokines, including TNF-α, IL-6, and IL-1β, which are released in elevated amounts which can contribute to various health issues.^36,37^ TNF-α and IL-1β protein levels in the agitated rats’ brains were considerably greater than those in the extract and control groups, according to the study’s results. The work by Page et al, which demonstrated that increased TK activity activates pro-inflammatory proteins and enhances the production of the TNF-α and IL-1β genes, supports this conclusion.^38^ Other studies reported an increase in the expression of TNF-α and IL-1β in the stressed rats.^39,40^

 In MsFs + CE extract group, there was a significant reduction in TNF-α protein levels in the offspring, while the decrease in the parents was not significant. Conversely, IL-1β protein levels showed a significant reduction in male parents of stress + CE extract group, but was not significant in the offspring. These findings align with the research by Hou et al., which showed that CE extract (150 mg/kg, IV) alleviated depression-like behaviors in rats subjected to chronic stress for 21 days and resulted in decreased levels of IL-1β and TNF-α cytokines.^33^ Quercetin, a key component of CE extract, is vital for its anti-inflammatory potential. It regulates various inflammatory signaling pathways,^35,41^ resulting in a significant reduction of inflammation by inhibiting pro-inflammatory cytokines, such as IL-1β and TNF-α.^42^ Our work’s examination of the indirect effects of stress and CE extract on offspring is among its most noteworthy features. The male progenitors and their offspring can only communicate via gametes. During the gametogenesis phase, adult rats were exposed to restraint stress, as detailed in the techniques section. They then came together to form the next generation. It had no effect on pregnant rats. Therefore, any effects and changes seen in this experiment should be attributed to variations in the gametes. Previous studies indicated that stress had an adverse effect on various parameters associated with semen quality, including sperm concentration, motility and morphology.^43,44^ Moreover, exposure to stress is associated with augmented oxidant production and long-term exposure to stressors may increase the generation of reactive oxygen species (ROS).^23,45^ The high ROS level 42 indicates that prolonged stress may cause an imbalance in the oxidant/antioxidant ratio, which increases lipid peroxidation. The strong antioxidant qualities of CE extract probably prevent or lessen the oxidative impact of stress on sperm quality in addition to partly counteracting the negative effects of stress on gametes. Future studies should assess potential epigenetic modifications in germ cells following chronic stress exposure.

## Conclusion

 Our results provide credence to the Persian medical theory on the spleen’s function under stress. CE may also be able to protect sperm and effectively reduce the damage that stress causes to the splenic and liver immune systems. It could also stop inflammatory responses in the brain. It is important to keep in mind that the next generation is also protected.

## Competing Interests

 The authors have no conflict of interest to declare.

## Data Availability Statement

 Data are available upon request.

## Ethical Approval

 This work was approved by the local ethic committee at the Zanjan University of Medical Sciences with following code: IR.ZUMS.AEC.1401.006.

## References

[1] Yi WJ, Kim TS (2017). Melatonin protects mice against stress-induced inflammation through enhancement of M2 macrophage polarization. Int Immunopharmacol.

[2] Bandegi AR, Rashidy-Pour A, Vafaei AA, Ghadrdoost B (2014). Protective effects of Crocus sativus L extract and crocin against chronic-stress induced oxidative damage of brain, liver and kidneys in rats. Adv Pharm Bull.

[3] Kurniawan DW, Jajoriya AK, Dhawan G, Mishra D, Argemi J, Bataller R (2018). Therapeutic inhibition of spleen tyrosine kinase in inflammatory macrophages using PLGA nanoparticles for the treatment of non-alcoholic steatohepatitis. J Control Release.

[4] Amini R, Asle-Rousta M, Aghazadeh S (2020). Hepatoprotective effect of limonene against chronic immobilization induced liver damage in rats. NaunynSchmiedebergs Arch Pharmacol.

[5] Sardari S, Mobaiend A, Ghassemifard L, Kamali K, Khavasi N (2021). Therapeutic effect of thyme (Thymus vulgaris) essential oil on patients with COVID-19: a randomized clinical trial. J Adv Med Biomed Res.

[6] Bale TL (2015). Epigenetic and transgenerational reprogramming of brain development. Nat Rev Neurosci.

[7] Li YC, Zheng XX, Xia SZ, Li Y, Deng HH, Wang X (2020). Paeoniflorin ameliorates depressive-like behavior in prenatally stressed offspring by restoring the HPA axis- and glucocorticoid receptor- associated dysfunction. J Affect Disord.

[8] Shah DR, Shah RR, Morganroth J (2013). Tyrosine kinase inhibitors: their on-target toxicities as potential indicators of efficacy. Drug Saf.

[9] Feng B, Xu JJ, Bi YA, Mireles R, Davidson R, Duignan DB (2009). Role of hepatic transporters in the disposition and hepatotoxicity of a HER2 tyrosine kinase inhibitor CP-724,714. Toxicol Sci.

[10] Liu ZJ, Wang YL, Li QL, Yang L (2018). Improved antimelanogenesis and antioxidant effects of polysaccharide from Cuscuta chinensis Lam seeds after enzymatic hydrolysis. Braz J Med Biol Res.

[11] Wang Z, Fang JN, Ge DL, Li XY (2000). Chemical characterization and immunological activities of an acidic polysaccharide isolated from the seeds of Cuscuta chinensis Lam. Acta Pharmacol Sin.

[12] Thomas S, Shrikumar S, Velmurugan C, Ashok Kumar BS (2015). Evaluation of anxioltic effect of whole plant of “Cuscutareflexa”. World J Pharm Sci.

[13] Jang JY, Kim HN, Kim YR, Choi YH, Kim BW, Shin HK (2012). Aqueous fraction from Cuscuta japonica seed suppresses melanin synthesis through inhibition of the p38 mitogen-activated protein kinase signaling pathway in B16F10 cells. J Ethnopharmacol.

[14] Donnapee S, Li J, Yang X, Ge AH, Donkor PO, Gao XM (2014). Cuscuta chinensis Lam: a systematic review on ethnopharmacology, phytochemistry and pharmacology of an important traditional herbal medicine. J Ethnopharmacol.

[15] Kong X, Zheng K, Tang M, Kong F, Zhou J, Diao L, et al. Prevalence and factors associated with depression and anxiety of hospitalized patients with COVID-19. medRxiv [Preprint]. April 5, 2020. Available from: https://www.medrxiv.org/content/10.1101/2020.03.24.20043075v2.

[16] Kılıçkaya Selvi E, Turumtay H, Demir A, Akyüz Turumtay E (2018). Phytochemical profiling and evaluation of the hepatoprotective effect of Cuscuta campestris by high-performance liquid chromatography with diode array detection. Anal Lett.

[17] Chabra A, Monadi T, Azadbakht M, Haerizadeh SI (2019). Ethnopharmacology of Cuscutaepithymum: a comprehensive review on ethnobotany, phytochemistry, pharmacology and toxicity. J Ethnopharmacol.

[18] Deepa P, Bae HJ, Park HB, Kim SY, Choi JW, Kim DH (2020). Dracocephalummoldavica attenuates scopolamine-induced cognitive impairment through activation of hippocampal ERK-CREB signaling in mice. J Ethnopharmacol.

[19] Ghassemifard L, Sardari S, Safari H, Ramezanikhah H, Khavasi N (2022). Assessment of the antioxidant activity of the extract, cold press, and n-hexane oils and in vitro cytotoxic effects of Capparis spinosa L seed on SH-SY5Y cancer cell lines. J Med Plants.

[20] Nakhjiri E, Saboory E, Roshan-Milani S, Rasmi Y, Khalafkhani D (2017). Effect of prenatal restraint stress and morphine co-administration on plasma vasopressin concentration and anxiety behaviors in adult rat offspring. Stress.

[21] Wu F, Zhao S, Yu B, Chen YM, Wang W, Song ZG (2020). A new coronavirus associated with human respiratory disease in China. Nature.

[22] Gao X, Li Y, Wang H, Li C, Ding J (2017). Inhibition of HIF-1α decreases expression of pro-inflammatory IL-6 and TNF-α in diabetic retinopathy. Acta Ophthalmol.

[23] Liu JB, Li ZF, Lu L, Wang ZY, Wang L (2022). Glyphosate damages blood-testis barrier via NOX1-triggered oxidative stress in rats: long-term exposure as a potential risk for male reproductive health. Environ Int.

[24] Amini M, Saboory E, Pourheydar B, Bagheri M, Naderi R (2020). Involvement of endocannabinoid system, inflammation and apoptosis in diabetes induced liver injury: role of 5-HT3 receptor antagonist. Int Immunopharmacol.

[25] El-Tahawy N, Ali AH (2021). Swimming exercise ameliorates the chronic immobilization stress-induced alterations in spleen and splenic T-cell population in adult male albino rats: histological and immunohistochemical study. Egypt J Histol.

[26] Wei Y, Wang T, Liao L, Fan X, Chang L, Hashimoto K (2022). Brain-spleen axis in health and diseases: a review and future perspective. Brain Res Bull.

[27] Xia L, Zhang B, Sun Y, Chen B, Yu Z (2021). Analysis of Syk/PECAM-1 signaling pathway in low shear stress induced atherosclerosis based on ultrasound imaging. Comput Methods Programs Biomed.

[28] Hu Y, Metzler B, Xu Q (1997). Discordant activation of stress-activated protein kinases or c-Jun NH2-terminal protein kinases in tissues of heat-stressed mice. J Biol Chem.

[29] Pourhadi M, Niknam Z, Ghasemi R, Soufi Zomorrod M, Niazi V, Faizi M (2022). Cuscutaepithymum Murr crude extract pre-conditioning protects C6 cells from L-glutamate-induced neurotoxicity. BMC Complement Med Ther.

[30] Gangarde P, Bhatt S, Pujari R (2025). Assessment of neuroprotective potential of Cuscutareflexain aluminium chloride-induced experimental model of Alzheimer’s disease: in vitro and in vivo studies. J Trace Elem Med Biol.

[31] Sudam VS, Potnuri AG, Subhashini NJP (2017). Syk - GTP RAC-1 mediated immune-stimulatory effect of Cuscutaepithymum, Ipomoea batata and Euphorbia hirta plant extracts. Biomed Pharmacother.

[32] Rajaee F, Hadigol T, Salehi Z, Farzam A. The effects of crowding stress on histological structure of mouse spleen. J Sabzevar Univ Med Sci 2014;21(1):207-15. [Persian].

[33] Hou L, Yang L, Zhu C, Miao J, Zhou W, Tang Y (2023). Cuscutae semen alleviates CUS-induced depression-like behaviors in mice via the gut microbiota-neuroinflammation axis. Front Pharmacol.

[34] Lin HB, Lin JQ, Lin JQ, Lu N, Yi XY (2003). [Comparative study on immune enhancement effects of four kinds of dodder seeds in Shandong province]. Zhong Xi Yi Jie He Xue Bao.

[35] Lee MS, Chen CJ, Wan L, Koizumi A, Chang WT, Yang MJ (2011). Quercetin is increased in heat-processed Cuscuta campestris seeds, which enhances the seed’s anti-inflammatory and anti-proliferative activities. Process Biochem.

[36] Liao JC, Chang WT, Lee MS, Chiu YJ, Chao WK, Lin YC (2014). Antinociceptive and anti-inflammatory activities of Cuscuta chinensis seeds in mice. Am J Chin Med.

[37] Hashemi P, Ebrahimi L, Saboory E, Roshan-Milani S (2013). Effect of restraint stress during gestation on pentylenetetrazol-induced epileptic behaviors in rat offspring. Iran J Basic Med Sci.

[38] Page TH, Smolinska M, Gillespie J, Urbaniak AM, Foxwell BM (2009). Tyrosine kinases and inflammatory signalling. Curr Mol Med.

[39] de Michelis Mograbi K, Suchecki D, da Silva SG, Covolan L, Hamani C (2020). Chronic unpredictable restraint stress increases hippocampal pro-inflammatory cytokines and decreases motivated behavior in rats. Stress.

[40] Pezeshki-Nia S, Asle-Rousta M, Mahmazi S (2020). Spinacia oleracea L extract attenuates hippocampal expression of TNF-α and IL-1β in rats exposed to chronic restraint stress. Med J Islam Repub Iran.

[41] Hosseini A, Rasmi Y, Rahbarghazi R, Aramwit P, Daeihassani B, Saboory E (2019). Curcumin modulates the angiogenic potential of human endothelial cells via FAK/P-38 MAPK signaling pathway. Gene.

[42] Nooreen Z, Siddiqui NA, Wal P, Shukla A, Verma RS, Ahmad A (2023). Evaluation of Cuscutareflexa seed essential oil on TPA-induced inflammation in mice. J King Saud Univ Sci.

[43] Mahmoodkhani M, Saboory E, Roshan-Milani S, Azizi N, Karimipour M, Sayyadi H (2018). Pre-pregnancy stress suppressed the reproductive systems in parents and changed sex ratio in offspring. J Appl Biomed.

[44] Azizi N, Heidari M, Saboory E, Abdollahzade N, Roshan-Milani S (2024). Investigating the effect of parental pre-gestational stress on ethological parameters in male rat offspring. J Vet Behav.

[45] Mahmoodkhani M, Saboory E, Roshan-Milani S, Azizi N, Karimipour M, Rasmi Y (2018). Pregestational stress attenuated fertility rate in dams and increased seizure susceptibility in offspring. Epilepsy Behav.

